# The Second Look after Fights: Why Wounds Might Not Only Be Superficial

**DOI:** 10.1155/2016/9063621

**Published:** 2016-03-17

**Authors:** Egemen Küçük, Alauddin Kochai, Ümit Fikret Onur, Yasemin Yıldız Kirazaldı, Ali Murat Başak

**Affiliations:** ^1^Department of Emergency Medicine, Sakarya University Training and Research Hospital, Adapazarı, 54100 Sakarya, Turkey; ^2^Department of Orthopedics and Traumatology, Sakarya University Training and Research Hospital, Adapazarı, 54100 Sakarya, Turkey; ^3^Department of Emergency Medicine, Sakarya University Faculty of Medicine, Adapazarı, 54100 Sakarya, Turkey; ^4^Department of Orthopedics and Traumatology, Sakarya University Faculty of Medicine, Adapazarı, 54100 Sakarya, Turkey

## Abstract

*Introduction*. We present a case of intraosseous foreign body penetration due to knife attack and its emergency service management.* Case*. Seventeen-year-old patient was admitted to the emergency department with a knife cut over the right knee. In the local wound exploration during the extension position of the knee, deep tissue penetration was not observed. Therefore, the patient was discharged after a primary wound saturation without any radiographic evaluation. During the second admission, the detailed anamnesis revealed that the injury occurred while the knee was in the flexion and the radiographic examination displayed a broken knifepoint in the sagittal plane of the femur's medial patellar region penetrated in the intraosseous tissue.* Conclusion*. Intraosseous foreign body cases due to the knife attacks are quite rare. There is no algorithm, indicating the necessity of radiographic examination in the stab wounds. Local wound exploration of stab wounds should be done in accordance with the mechanism of injury.

## 1. Introduction

Some mechanisms that can lead to the penetration of foreign bodies into the organism are traffic accidents, explosions, occupational accidents, gunshot injuries, and operations [1]. Majority of the reported foreign bodies are located in the soft tissues, particularly in the subcutaneous tissue [2]. Reports of intraosseous foreign bodies are rare and more common after surgical treatment with orthopedic implants [3, 4]. Intraosseous foreign bodies due to the knife attacks are rather rarely encountered. There is only one case in the literature [5].

In this study, intraosseous foreign body penetration due to knife attack and its emergency service management are reported.

## 2. Case

**Figure 1:**
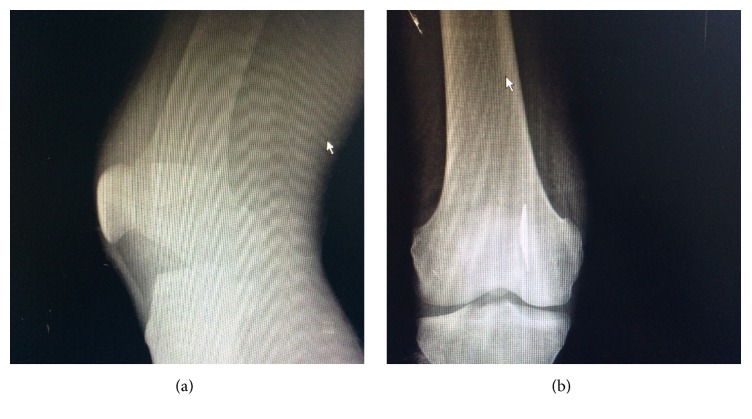
(a) Lateral radiographic examination of the knee. (b) Anteroposterior radiographic examination of the knee.

Seventeen-year-old male patient, who had a cut over the right knee due to a knife attack, was admitted to the emergency department one hour after the event. During the examination, his measured blood pressure was 110/70 mmHg, heart rate 82/minute, and oxygen saturation 99%. A longitudinally located, nearly 3 centimeters long cut with smooth margins on the medial part of the right knee was observed. Peripheral pulse was detectable, motor and sensory examination was normal, and motility of extremity was not limited. The local wound exploration committed during the extension of the right knee did not show any sign of the deep penetration. The lesion was evaluated as a simple superficial cut and the patient was discharged after the tetanus prophylaxis, wound care, and saturation. Two days after the discharge, the patient was readmitted to the emergency department with the complaints of persistent pain on the right knee, swelling, and mild redness. During the second examination, we found out that the flexion and extension of the right knee were painful and there was mild hyperaemia over the right knee around the sutured lesion. Vital signs of the patient were normal, blood pressure was 100/70 mmHg, heart rate was 75/minute, oxygen saturation was 99%, and fever was 37°C. The detailed anamnesis of the patient revealed that he got this stab wound while he attempted to hit the attacker's abdomen with his bent right knee. In the bidirectional radiographic examination, a broken knifepoint (nearly 3 centimeters long) behind the right patella in the medial region of the femur, on the sagittal plane with intraosseous penetration, was detected (Figures 1(a) and 1(b)).

Peripheral pulse, sensory, and motor examination of the patient were normal. An antibiotic treatment (intravenous 1 g ampicillin-sulbactam and 80 mg gentamycin) was started and an orthopedic consultation was requested.

**Figure 2:**
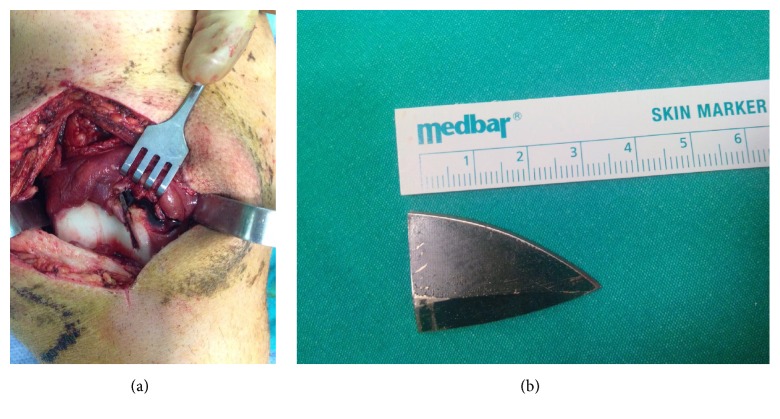
(a) Intraoperative appearance of the knifepoint. (b) Postoperative appearance of the knifepoint.

The patient was taken to the operating room for removal of the foreign body. In the operating room, following the appropriate staining and draping, the bottom of the medial retinaculum accessed with a longitudinal incision through the medial site of the patella and the foreign body was detected. The blade was noted sagittally through the medial patellar area of the femur (Figures 2(a) and 2(b)).

Pliers were used to remove the knifepoint, without incident. Area of the operation was irrigated with 5000 mL normal saline, and the wound was closed loosely. Intraoperative X-ray showed no retained foreign body. The patient received intravenous antibiotics (cefazolin, gentamycin) for 48 hours after surgery. The patient remained neurovascularly intact and had no appreciable deficits. At the third day of the operation, the patient was discharged with oral cefazolin treatment. An orthopedic examination in the outpatient clinic was also recommended.

## 3. Discussion

Violence related injuries are now regarded as a serious problem. Majority of victims are males between the ages of fifteen and forty-five [6]. Injury rates with a knife are relatively low among the violence related injuries. Most of the cases are males (85–95%) and the mean age is thirty-two. Stab wounds are mostly located in the torso (thorax, abdomen, and buttocks), and the rarest area seen is lower extremities [7]. The youngish male we are presenting had a knife wound in his right lower extremity. A stab wound to the extremity can damage structures deep to the skin such as blood vessels, nerves, or tendons and additionally can carry bacteria or foreign material deep into the wound and cause infections [8]. At the first emergency department admission, there were no hard or soft signs of arterial injury [9] and loss of mobility in our patient; additionally, motor and sensory examination was normal.

During the first admission to the emergency department, the local wound exploration was done on the extension position of the knee and no sign of any deep penetration was observed. The main reason for this is that the injury occurred during the full flexion of the knee. The patella is the largest sesamoid bone in the body and located within the tendon of the quadriceps femoris muscle. During the flexion of the knee, patella moves down vertically almost two times of its length (nearly 8 centimeters). At 135 degrees of flexion (during the squatting position), the patella slips into the intercondylar notch. The patellar facets of the femur exhibit an extensive contact area with both the patella and the broad posterior surface of the quadriceps tendon. In this position, patellar facets of the femur will be available to the trauma. In the full extension, patella partially covers the patellar facet of the femur [10, 11]. As in the reported case, the signs of the deep penetration may be overlooked, if the penetrating injury occurred during the full flexion of the knee and the local wound exploration is done on the extension position of the knee, while in the local wound examination of the freely moveable joints like knee, the position of the joint during the wounding should be kept in mind.

Screening of the literature displayed only one study reporting on intraosseous foreign body penetration due to the knife attack [5]. There is only limited information in the literature about the emergency service management of the patients with an intraosseous foreign body. We could not identify any information pointing to the necessity of a routine direct radiographic examination of the wounds that occurred due to the knife attack [12, 13]. In the presented case, as no sign of deep penetration during the local wound exploration was observed, the lesion is interpreted as a superficial cut and no radiographic examination was requested. There is no conclusive evidence supporting prophylactic antimicrobial use in the management of small soft tissue extremity trauma and simple lacerations [14]. Therefore, wound care, saturation, and tetanus prophylaxis were performed, but no antibiotic treatment was initiated.

Intraosseous foreign body cases due to the knife attack are quite rare. There is no algorithm indicating direct radiographic examination in the stab wounds. In order to determine the deep tissue penetration of knife cuts during the examination, the details of the injury (how the trauma occurred) should be questioned during the anamnesis and the local wound exploration should be done accordingly.
